# Effects of lavender essential oil inhalation aromatherapy on postoperative sleep quality in patients with intracranial tumors: a randomized controlled trial

**DOI:** 10.3389/fphar.2025.1584998

**Published:** 2025-08-04

**Authors:** Yang Liu, Yanmei Dong, Xin Wang, Yeqin Huang, Fan Wu, Fei Xia, Hongtong Bai, Hui Li, Lei Shi, Baoguo Wang

**Affiliations:** ^1^Department of Anesthesiology, Sanbo Brain Hospital, Capital Medical University, Beijin, China; ^2^State Key Laboratory of Plant Diversity and Specialty Crops, Institute of Botany, Chinese Academy of Sciences, Beijing, China; ^3^ China National Botanical Garden, Beijing, China; ^4^Department of Chemistry “G. Ciamician”, University of Bologna, Bologna, Italy

**Keywords:** lavender essential oil, intracranial tumor, sleep quality, perioperative neurocognitive disorders, anxiety

## Abstract

**Purpose:**

To investigate the effects of lavender essential oil (LEO) on postoperative sleep quality and perioperative neurocognitive disorders (PNDs) in patients with intracranial tumors.

**Patients and methods:**

This study was a randomized controlled trial in which all patients were randomly assigned to either the control group, which received no intervention, or the experimental group, which received LEO. Postoperative sleep quality was assessed using a dedicated sleep monitor. The PNDs were evaluated by the Confusion Assessment Method for the Intensive Care Unit (CAM-ICU) and the Mini-Mental State Examination (MMSE), using education-adjusted cutoffs recommended by the latest Chinese Dementia Guidelines (2023).

**Results:**

Compared with the control group, the total sleep duration and the deep sleep duration were significantly longer in the experimental group only on the fourth postoperative day (418.48 ± 21.95 vs. 389.57 ± 49.29, P = 0.019; 95.10 ± 19.98 vs. 66.86 ± 32.69, P = 0.002). The experimental group exhibited significantly shorter sleep latency compared with the control group (13.24 ± 8.46 vs. 28.62 ± 19.86; p = 0.002). Additionally, the apnea-hypopnea index and frequency of awakenings were lower in the experimental group (14.05 ± 9.85 vs. 21.00 ± 10.78; p = 0.035; 2.67 ± 1.32 vs. 5.05 ± 2.97; p = 0.002). The duration of postoperative delirium was shorter in the experimental group compared with the control group (2.00 ± 0.82 vs. 3.80 ± 1.30; p = 0.048). On the seventh postoperative day, participants in the experimental group had lower anxiety scores than those in the control group (3.38 ± 2.27 vs. 6.14 ± 5.43; p = 0.038).

**Conclusion:**

LEO inhalation aromatherapy could effectively improve postoperative sleep quality, particularly on the fourth postoperative day. It also positively impacted anxiety and reduced the duration of postoperative delirium.

**Clinical Trial Registration:**

https://www.chictr.org.cn/index.html, identifier ChiCTR2300073091.

## Introduction

Intracranial tumors are common diseases in neurosurgery, which can impair brain function, significantly affect patients’ physical and mental health, and, in severe cases, become life-threatening (Banan and Hartmann, 2017). Currently, surgical intervention remains the primary treatment for intracranial tumors; however, it is often accompanied by postoperative neurocognitive dysfunction, reduced self-care capacity, and prolonged hospitalization, all of which impose a remarkable psychological burden on patients during their hospital stay (Qiu et al., 2022). A comprehensive systematic review published in 2023 examined sleep disturbances in adults with primary brain tumors (PBTs). It found that the prevalence of sleep disturbances varied widely across studies, with estimates ranging from 17% to 81.8%, depending on the assessment tools and patient populations involved (Martin et al., 2023).

Increasing evidence demonstrated that postoperative sleep disturbances are associated with cognitive impairment, aggravated postoperative pain, anxiety and depression, increased complications, and delayed postoperative recovery (Roggenbach et al., 2014; Devinney et al., 2022; Shakya et al., 2019). Perioperative neurocognitive disorders (PNDs) refer to a spectrum of cognitive impairments occurring during the perioperative period, primarily including postoperative delirium and delayed neurocognitive recovery (Kong et al., 2022). PNDs are associated with increased healthcare costs, morbidity, and mortality. Consequently, both postoperative sleep disturbances and PNDs represent critical challenges that must be addressed to enhance short- and long-term clinical outcomes. Previous studies have demonstrated that postoperative sleep disruptions may induce neuroinflammation, impair hippocampal synaptic plasticity, and alter cerebrospinal fluid biochemistry, ultimately contributing to cognitive decline (Martin et al., 2023; Wadhwa et al., 2017; Rasmussen et al., 2018). As the goal of perioperative medicine extend beyond survival to the patient’s physical and psychological well-being, the management of postoperative sleep disorders and PNDs has become increasingly important. Therefore, identifying effective and safe strategies to improve sleep quality during the perioperative period is of great significance for optimizing clinical outcomes.

Strategies to enhance postoperative sleep quality include both pharmacological and non-pharmacological interventions. Although pharmacological agents may be effective, they mainly accompany by adverse effects, such as respiratory depression and disruption of normal sleep architecture, and may pose risks of tolerance or dependency (Young et al., 2009). Meanwhile, non-pharmacological interventions, such as noise reduction, the use of eye masks, music therapy, and other complementary techniques, have been explored to improve perioperative sleep quality. However, due to significant individual variability in response, no universally effective non-drug intervention has yet been established. Inhalation aromatherapy, involving the inhalation of essential oils, has increasingly attracted attention as a non-pharmacological therapy because of its minimal side effects, ease of application, and potential to alleviate diverse physical and psychological symptoms (Devinney et al., 2022; Shakya et al., 2019; Lucena et al., 2024). Among various essential oils, lavender essential oil (LEO) is one of the most widely used in aromatherapy and is known for its anti-inflammatory, anxiolytic, antidepressant, and sleep-promoting properties, as well as its efficacy in relieving migraines and insomnia (Kong et al., 2022; Lucena et al., 2024; Luo and Jiang, 2022). A randomized controlled trial by Tayebi et al. (2015) investigated the effects of LEO inhalation on depression, anxiety, and stress in hemodialysis patients. Participants inhaled lavender oil during dialysis sessions over 4 weeks. The study found significant reductions in depression and stress levels in the intervention group compared to the control group. Furthermore, studies have demonstrated that inhalation aromatherapy using LEO significantly reduces stress, anxiety, and discomfort, while also improving vital signs in burn patients (Chen et al., 2022; Akkaya et al., 2024; Ghavami et al., 2022; Ebrahimi et al., 2021). Notably, the anxiolytic effects of lavender oil may be more remarkable in female patients compared with male patients (Ebrahimi et al., 2021). Additionally, for cases experiencing insomnia, single-scent lavender inhalation has been reported to be more effective than mixed-aroma inhalation methods (Cheong et al., 2021; Cheng et al., 2022).

Although recent evidence suggests that the therapeutic potential of LEO in managing various physiological and psychological disorders, its efficacy in alleviating postoperative sleep disturbances in patients with intracranial tumors remains elusive and warrants further investigation. Moreover, the therapeutic properties of LEO are closely linked to its chemical composition. The primary bioactive constituents (linalool and linalyl acetate) may enhance perioperative sleep quality by entering the circulatory system through inhalation and modulating the GABAergic, cholinergic, histaminergic, and monoaminergic pathways in the limbic system (Xu et al., 2023). Therefore, this trial aimed to investigate the impact of LEO on postoperative sleep quality in patients with intracranial tumors.

## Materials and methods

### Participants

A total of 42 inpatients scheduled for elective craniotomy for intracranial tumors were recruited from Sanbo Brain Hospital between July and October 2023. Written informed consent was obtained from all participants or their legal representatives prior to enrollment in the study.

All participants met the following inclusion criteria (Banan and Hartmann, 2017): Age ≥18 years (Qiu et al., 2022), Patients with sleep disorders (Pittsburgh Sleep Quality Index score >5) (Martin et al., 2023), Patients voluntarily participating in the clinical study and have signed informed consent.

The exclusion criteria were as follows (Banan and Hartmann, 2017): History of psychiatric disorders, or currently taking psychiatric medication (Qiu et al., 2022); History of olfactory impairment in the past month (Martin et al., 2023); History of allergies or eczema (Roggenbach et al., 2014); Participating in any other intervention studies related to this research.

### Study design

This study was designed as a randomized controlled trial. Ethical approval was granted by the institutional ethics committee (Approval No. SBNK-YJ-2023-015-01), and the trial was registered in the Chinese Clinical Trial Registry (https://www.chictr.org.cn/index.html, Registration No. ChiCTR2300073091).

Patients were randomly allocated to either the experimental group or the control group using block randomization with a block size of four. The randomization sequence was generated using a computer-based random number generator by an independent statistician who was not involved in patient recruitment or data collection. Allocation concealment was ensured using sequentially numbered, opaque, sealed envelopes (SNOSE), which were opened only after the patient consented and was enrolled in the study. The experimental group received inhalation aromatherapy with LEO. Specifically, five drops of 10% LEO were applied to the cotton pad of a nasal patch, which was positioned near the nasolabial fold. The intervention was administered nightly for seven consecutive days following surgery, from 20:00 to 08:00. The control group received no intervention. On the day of admission, patients were evaluated for sleep disorders, anxiety, depression, pain levels, daily activities, and cognitive function using the Pittsburgh Sleep Quality Index (PSQI), the Hospital Anxiety and Depression Scale (HADS), the Numerical Pain Rating Scale (NPRS), the Activities of Daily Living (ADL) scale, and the Mini-Mental State Examination (MMSE), respectively.

LEO was selected for its established anxiolytic and sleep-promoting effects, with evidence suggesting a favorable safety profile and ease of use in clinical settings. The inhalation method via a nasal patch allowed for non-invasive, continuous nighttime delivery, which was well tolerated and easily incorporated into the standard postoperative care routine without disrupting medical procedures or patient rest. While challenges, such as patient variability in olfactory sensitivity and individual psychological response to aromatherapy may affect outcomes, this approach shows promise for broader implementation. The accessibility, low cost, and minimal training required for administration support the potential applicability of LEO aromatherapy in daily healthcare practice, especially in resource-limited or non-pharmacological care settings.

### Plant materials

The *Lavandula angustifolia* cultivar ‘Jingxun 2’ was used in this study. A voucher specimen (No. 02308796) is deposited in the Chinese National Herbarium, Institute of Botany, Chinese Academy of Sciences. Dried flowering spikes were ground to a coarse powder with an average particle size of approximately 2–3 mm before extraction. A total of 100 g of the powdered plant material was mixed with 1,000 mL of distilled water (a water-to-material ratio of 10:1, w/v) and subjected to steam distillation for 3 h using a Clevenger-type apparatus. The extracted LEOs were dried over anhydrous sodium sulfate and stored in amber glass bottles at 4°C until further use.

### Analysis of LEO by GC–MS

LEO was filtered and diluted in n-hexane at a ratio of 1:200 prior to analysis. Gas chromatography-mass spectrometry (GC–MS) was performed using a 7890A-7000B instrument (Agilent Technologies, Santa Clara, CA, United States) equipped with an HP-5MS capillary column (30 m × 250 μm × 0.25 μm, Agilent Technologies). The injector temperature was set at 250°C. A 1 μL aliquot of the sample was injected in split mode (split ratio 20:1). The oven temperature program was as follows: initial temperature of 60°C held for 4 min, ramped to 240°C at 6°C/min, then further increased to 280°C at 20°C/min. The transfer line temperature was maintained at 280°C. Helium served as the carrier gas at a constant flow rate of 2.25 mL/min. The MS operating conditions included an ionization energy of 70 eV, electron impact (EI) ion source temperature of 230°C, quadrupole temperature of 150°C, and a scan mass range of 40–700 u. Identification of LEO components was achieved by comparison with mass spectra from the NIST 17 library and retention index (RI) values. The retention index values were determined using n-alkane (C7-C40) hydrocarbons under the same conditions. The relative percentage of LEO components was determined based on the peak area.

LEO was carefully characterized for its chemical composition using GC-MS. This characterization is critical to ensure consistency in the chemical profile of LEO used in this study, as the composition can vary significantly depending on its method of extraction and origin. The LEO used in this study was also assessed for its suitability in multiple therapeutic modalities, including oral, topical, and inhalation routes. While this study primarily investigated inhalation aromatherapy, the broader safety and efficacy of LEO in these different applications have been explored in previous research (Saeed et al., 2023). However, it is noteworthy that the chemical composition of LEO might vary depending on the method of use (e.g., inhalation vs. oral ingestion) and could have different safety implications in each case. For instance, the inhalation of lavender oil has been reported to cause mild respiratory irritation in sensitive individuals, while no severe adverse effects have been reported in the literature for inhalation therapy when used as described in this study.

### Outcome measures

#### Primary outcome

The primary outcome of this study was the assessment of postoperative sleep quality using a wearable sleep monitoring device (SC-500TM; Boshi Linkage Technology, Beijing, China) for seven consecutive nights following surgery. This device uses multi-sensor fusion technology, including photoplethysmography (PPG), accelerometry, and temperature sensors, to non-invasively estimate sleep stages and respiratory parameters. Its algorithm has been validated against polysomnography in previous studies (Liu et al., 2023a; Liu et al., 2023b).

Eight sleep-related parameters were collected:

1. Total sleep duration (min)
2. Deep sleep duration (min) – defined by the algorithm as low movement and stable heart rate variability, corresponding to N3 sleep
3. Light sleep duration (min) – defined as periods of higher movement and variable autonomic activity, typically encompassing N1 and N2 stages
4. Rapid Eye Movement (REM) sleep duration (min)
5. Sleep latency (min) – the time from “lights off” to the first epoch of any sleep stage
6. Awakening frequency (counts per night) – defined as the number of awakenings lasting longer than 1 min
7. Apnea-Hypopnea Index (AHI, events/hour) – the number of apneas and hypopneas per hour of sleep
8. Sleep efficiency (%) – calculated as total sleep time divided by time in bed, expressed as a percentage

The proportions of each sleep stage (i.e., deep sleep, light sleep, and REM) relative to total sleep time were also recorded to evaluate sleep architecture. Data were considered valid only if nightly recordings exceeded 6 h in duration, with signal quality ≥85% based on the device’s built-in data integrity scoring system. Nights failing to meet these criteria were excluded from the final analysis.

#### Secondary outcome

Secondary outcome of this study was PNDs, which were mainly evaluated as postoperative delirium occurring up to 7 days after surgery, and postoperative cognitive dysfunction assessed at one and 3 months after surgery. The Confusion Assessment Method for the Intensive Care Unit (CAM-ICU) was used to assess postoperative delirium. The assessment was conducted twice daily (8:00 a.m.–10:00 a.m. and 6:00 p.m.–8:00 p.m., respectively), following the Richmond Agitation Sedation Scale (RASS) evaluation. The assessment was performed only in patients with RASS higher or equal to −3. The diagnosis of delirium is based on the presence of the first two criteria in the CAM-ICU flow sheet (i.e., acute or fluctuating mental status and inattention) plus at least one of the following criteria (altered level of consciousness and or disorganized thinking). The MMSE was used to assess postoperative cognitive dysfunction in the first and third postoperative months through telephone follow-up. The MMSE is the most common used cognitive screening tool in the world and can assess five dimensions including orientation, memory, language, recall, attention, and computation. Its total score was 30, with higher scores indicating better cognitive function. According to the 2023 Chinese Dementia Guidelines, education-adjusted MMSE cutoffs for cognitive dysfunction were applied as follows: <17 for illiterate individuals, <20 for primary school graduates, and <24 for those with secondary education or higher.

#### Clinical outcomes

Patients were assessed for pain using the NPRS and for nausea and vomiting using the Nausea and Vomiting Numerical Rating Scale (NVNRS) on the first and third postoperative days. The Hospitalized Anxiety and Depression Scale assessed anxiety and depression on the seventh postoperative day. We recorded all complications from the first to the seventh postoperative day by follow-up and graded the complications according to the Clavien-Dindo classification and calculated the Comprehensive Complication Index (CCI). Daily activities of patients were evaluated on the day of discharge and 3 months postoperatively using ADL scale. The laboratory test results, length of stay, and hospital costs were obtained from medical record system. Three months after surgery, subjective sleep quality was assessed through telephone follow-up using the PSQI.

### Sample size calculation

This study was designed as a randomized controlled clinical trial comparing the effect of LEO aromatherapy versus no intervention on postoperative sleep quality. The primary outcome was total sleep duration, a continuous variable. Sample size calculation was performed using PASS 2023 software (NCSS, LLC. Kaysville, Utah, United States), applying a two-sample t-test (two-sided) for comparison of means.

Based on data from a previous study, the control group was expected to have a total sleep duration of 403.6 min with a standard deviation (SD) of 51.6 min. The minimal clinically important difference was defined as an increase of 51.5 min in the experimental group. Assuming a significance level (α) of 0.05 and a power (1−β) of 90%, the required sample size per group was calculated as 17 participants.

The formula used for this calculation was:

$$n={\left(\frac{\left(Z_{1-\alpha /2}+Z_{1-\beta }\right)\;\sigma }{\delta }\right)}^{2}$$

where:

• Z_1−α/2_ = 1.96 (for α = 0.05),
• Z_1−β_ = 1.28 (for 90% power),
• σ = 51.6 (standard deviation),
• δ = 51.5 (expected difference between means).

Accounting for a potential dropout rate of 20%, a minimum of 21 participants per group was required, resulting in a total sample size of 42 participants.

### Statistical analyses

The normality of the variable’s distribution was analyzed using the Kolmogorov–Smirnov test. The data were presented as mean ± standard deviation, median (interquartile range), and frequency (percentage), as appropriate. The differences in categorical variables were assessed using the chi-square and Fisher’s exact tests. The differences in continuous variables were analyzed using the independent sample t-test or Mann-Whitney U test based on the data distribution. Sleep parameters with multiple valid daily recordings were averaged across the 7-day period for each patient prior to group comparison. A *P*-value < 0.05 was considered statistically significant in all analyses. All statistical analyses were performed using SPSS Statistics 25.0 (IBM Corp, Armonk, NY, United States).

Subgroup analyses were performed to explore sex-specific effects of LEO on postoperative outcomes. Differences between male and female participants within each group were evaluated using the same statistical methods as described above. Interaction terms between sex and group assignment were also assessed where appropriate.

## Results

### Participant characteristics

**FIGURE 1:**
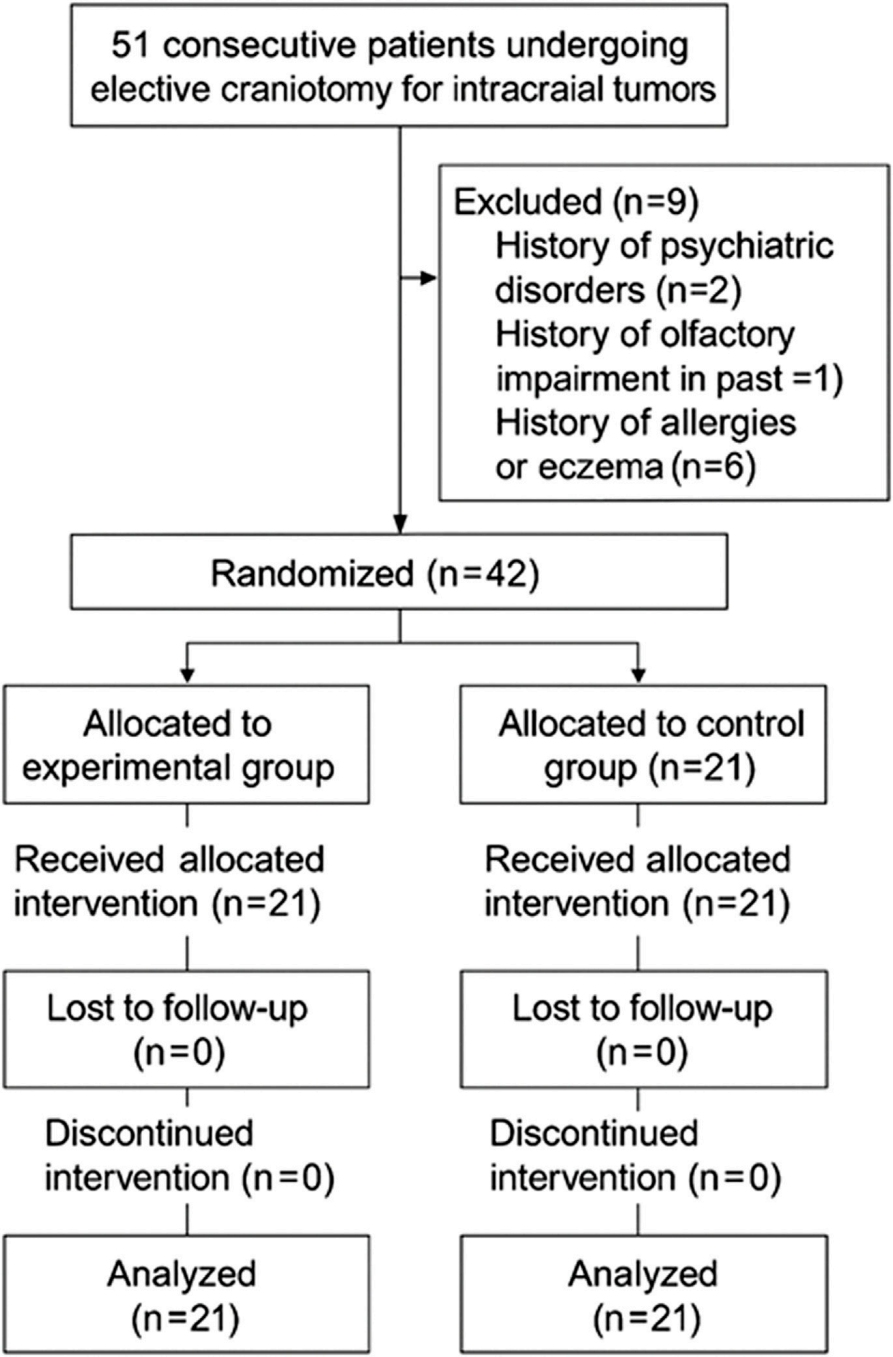
The study flowchart.

The flow chart of the whole experiment was shown in Figure 1. A total of 42 participants were randomly assigned into two groups between July and October 2023. The demographic and clinical information of the participants were shown in Table 1. There were no significant differences between the two groups in all variables.

**TABLE 1:** Baseline characteristics of participants.

Variables	Control group (n = 21)	Experimental group (n = 21)	χ^2^/t	P
Age	56.71 ± 9.48	55.29 ± 12.04	0.427	0.672
Gender (%)			0.404	0.525
male	7 (33.3)	9 (42.9)		
female	14 (66.7)	12 (57.1)		
BMI	26.30 (22.70, 27.70)	22.90 (22.00, 24.25)	−1.725	0.085
Years of education	9.00 (6.00, 15.00)	6.00 (3.00, 10.50)	−1.296	0.195
Drinking (%)	2 (9.5)	2 (9.5)	0.000	1.000
Smoking (%)	2 (9.5)	4 (19.0)	0.194	0.659
Hypertension (%)	7 (33.3)	4 (19.0)	1.109	0.292
Diabetes (%)	7 (33.3)	3 (14.3)	2.100	0.147
Sleep medications (%)	0 (0.0)	2 (9.5)	0.525	0.469
Tumor classification (%)			3.467	0.237
Meningioma	14 (66.7)	10 (47.6)		
Glioma	5 (23.8)	4 (19.0)		
Acoustic neuroma	2 (9.5)	7 (33.3)		
Tumor location (%)			4.783	0.316
Frontal lobe	7 (33.3)	5 (23.8)		
Parietal lobe	4 (19.0)	1 (4.8)		
Temporal lobe	5 (23.8)	5 (23.8)		
Occipital lobe	3 (14.3)	3 (14.3)		
Cerebellopontine angle	2 (9.5)	7 (9.5)		
Tumor area (%)			1.714	0.190
Supratentorial	16 (76.2)	12 (57.1)		
Infratentorial	5 (23.8)	9 (42.9)		
Malignant (%)	6 (28.6)	5 (23.8)	0.123	0.726
Tumor size	12.00 (8.00, 71.38)	16.68 (5.75, 55.32)	−0.491	0.623
CRP	0.74 (0.35, 6.74)	0.45 (0.22, 0.81)	−1.623	0.105
NLR	2.53 (1.56, 4.53)	1.87 (1.29, 2.41)	−1.547	0.122
MMSE	27.00 (26.00, 28.50)	28.00 (25.00, 30.00)	−0.853	0.394
HADSA	2.00 (0.00, 6.00)	3.00 (0.00, 4.00)	−0.077	0.939
HADSD	3.00 (0.00, 9.50)	2.00 (0.00, 4.00)	−0.960	0.337
PSQI	9.62 ± 3.38	9.86 ± 2.80	−0.249	0.805
NRS	0.00 (0.00, 2.00)	0.00 (0.00, 0.00)	−1.579	0.114
ADL	80.00 (80.00, 90.00)	85.00 (80.00, 90.00)	−1.502	0.133
Duration of surgery	315.00 ± 123.26	331.43 ± 103.16	−0.468	0.642
Postoperative analgesics (%)			4.492	0.097
Dezocine	14 (66.7)	14 (66.7)		
Sufentanil	2 (9.5)	6 (28.6)		
Sufentanil and Dezocine	5 (23.8)	1 (4.8)		

Notes: Abbreviations: BMI, body mass index; CRP, C-reactive protein; NLR, neutrophil-lymphocyte ratio; MMSE, mini-mental state examination; HADSA, hospital anxiety and depression scale-anxiety; HADSD, hospital anxiety and depression scale-depression; PSQI, pittsburgh sleep quality index; NRS, numerical rating scale; ADL, activities of daily living.

### Chemical compositions of lavender essential oil

**FIGURE 2:**
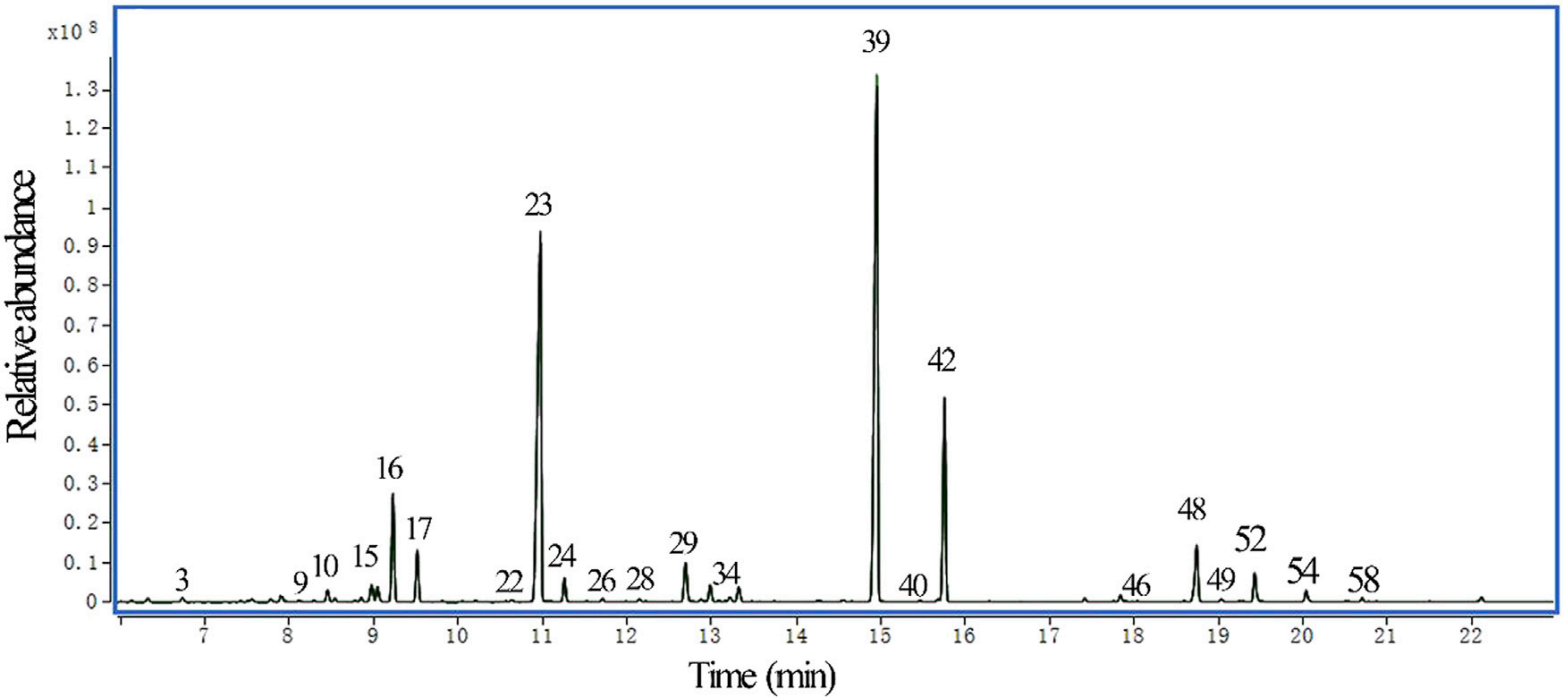
GC-MS chromatogram of LEO. Each peak corresponds to a volatile compound identified and numbered (Supplementary Table S1). The y-axis represents relative abundance (in arbitrary units), scaled by a factor of 10^8^, which reflects the ion current intensity detected for each compound relative to the most intense peak (compound 39, linalyl acetate). The x-axis indicates retention time in minutes.

The chemical composition of LEO was analyzed by GC-MS, revealing the presence of 60 compounds with individual concentrations exceeding 0.01%. The major constituents were linalyl acetate (34.50%), linalool (27.85%), and lavandulol acetate (10.74%). The chromatographic separation of these components is shown in Figure 2 and Supplementary Table S1. They corresponded to the most significant compounds as reported by previous studies on LEOs [28]. Other high abundant compounds were β-ocimene (5.42%), caryophyllene (3.51%), trans-β-ocimene (2.45%), endo-borneol (2.42%), farnesene (1.55%), 1-octen-3-yl-acetate (1.19%), β-phellandrene (1.04%).

### Postoperative sleep quality

**FIGURE 3:**
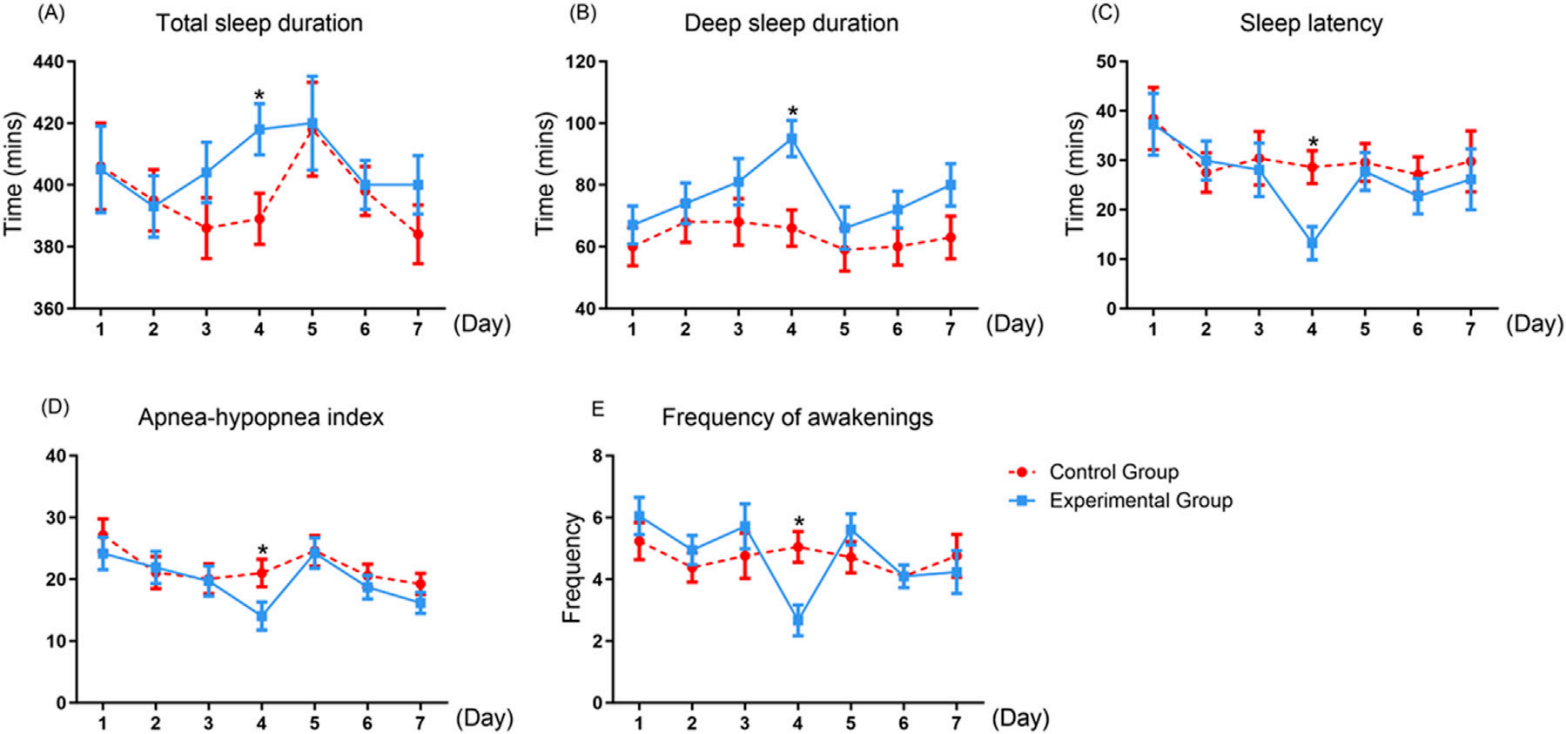
The differences in postoperative sleep quality parameters between two groups. Values are presented as mean ± standard error (SE) from twenty-one replicates. **(A)** Total sleep duration; **(B)** Deep sleep duration; **(C)** Sleep latency; **(D)** Apnea–hypopnea index; **(E)** Frequency of awakenings. Asterisk (*) indicates significant differences (*P < 0.05).

We recorded sleep quality parameters in the two groups using a dedicated sleep device for 7 days after surgery, and analyzed the differences in the 7-day mean values of each parameter between the two groups in Figure 3. Compared with the control group, the total sleep duration and the deep sleep duration were significantly longer in the experimental group only on the fourth postoperative day (418.48 ± 21.95 vs. 389.57 ± 49.29, P = 0.019; 95.10 ± 19.98 vs. 66.86 ± 32.69, P = 0.002). Although improvements in sleep parameters were found across several nights in the experimental group, statistically significant differences between groups were only found on the fourth postoperative day. On other days, while trends toward improved sleep were noted, the differences did not reach statistical significance. These findings suggest a potential delayed or cumulative effect of repeated lavender essential oil exposure, which may require several nights to exert a measurable impact on postoperative sleep quality. The sleep latency was shorter in the experimental group (13.24 ± 8.46 vs. 28.62 ± 19.86; *P* = 0.002). Besides, the apnea-hypopnea index and frequency of awakenings were smaller in the experimental group (14.05 ± 9.85 vs. 21.00 ± 10.78, *P* = 0.035; 2.67 ± 1.32 vs. 5.05 ± 2.97, *P* = 0.002).

### Perioperative neurocognitive disorder

The effects of LEO on the perioperative neurocognitive disorder were shown in Table 2. Among the two groups, the duration of postoperative delirium in the experimental group was shorter compared with the control group (2.00 ± 0.82 vs. 3.80 ± 1.30; *P* = 0.048). No significant difference was observed in the incidence, time of onset, and subtype of postoperative delirium. Furthermore, there was no significant difference in long-term postoperative cognitive function assessed by MMSE at 1 month and 3 months after surgery between the two groups.

**TABLE 2:** Perioperative neurocognitive disorders between control group and experimental group.

Variables	Control group (n = 21)	Experimental group (n = 21)	χ^2^/t	P
Postoperative delirium				
Incidence	5 (23.8%)	4 (19.0%)	0.000	1.000
Time of onset			1.704	0.683
1	1 (50.0)	1 (50.0)		
2	4 (66.7)	2 (33.3)		
4	0 (0.0)	1 (100.0)		
Time of duration	3.80 ± 1.30	2.00 ± 0.82	2.393	0.048
Subtype			1.457	0.714
Hyperactive	1 (50.0)	1 (50.0)		
Hypoactive	3 (75.0)	1 (25.0)		
Mixed	1 (33.3)	2 (66.7)		
Postoperative cognitive dysfunction				
MMSE-1month	27.00 (25.00, 27.50)	27.00 (25.00, 29.00)	−1.109	0.268
MMSE-3month	26.00 (24.00, 26.00)	26.00 (24.50, 28.00)	−1.542	0.123

Notes: Abbreviations: MMSE, mini-mental state examination.

### Clinical outcomes

**FIGURE 4:**
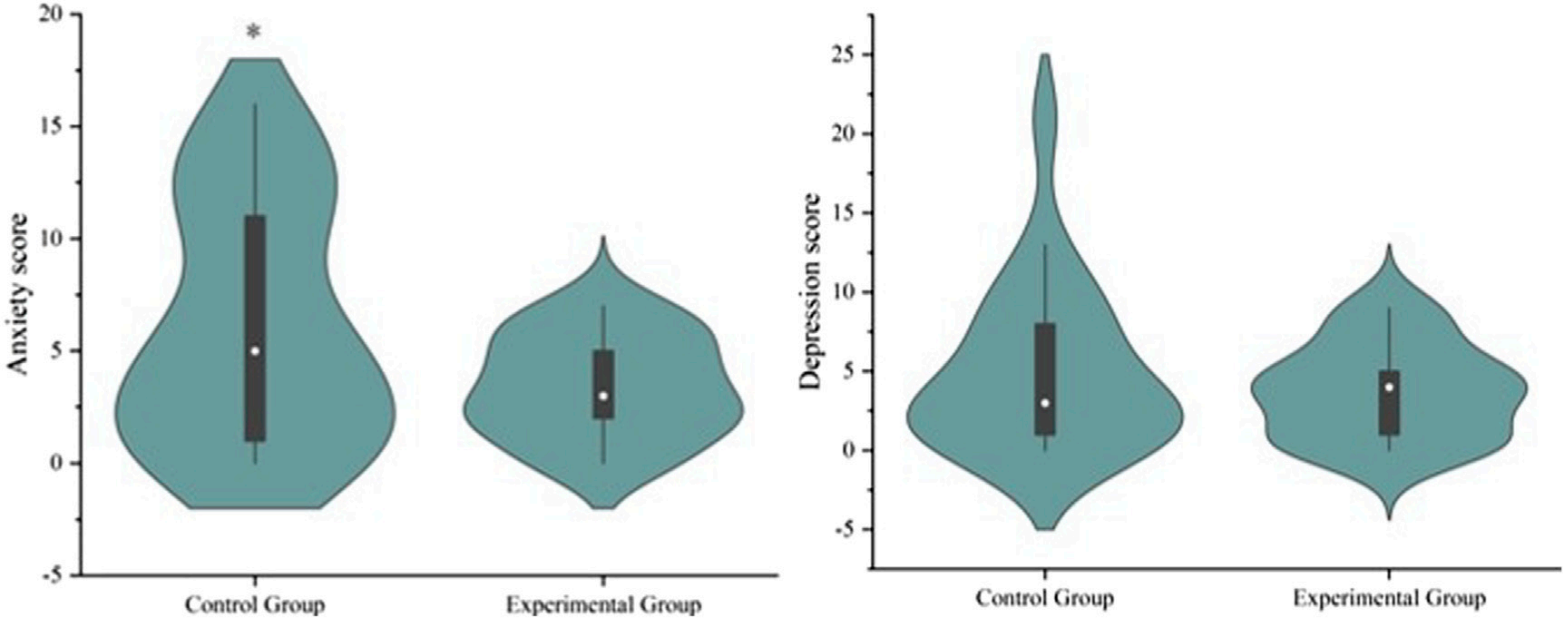
Analysis of anxiety scores in two groups. Values are expressed as mean ± standard error (SE) based on twenty-one replicates. Asterisk (*) indicates significant differences (*P < 0.05).

In our study, we found that the participants received lavender essential oil could significantly reduce the anxiety scores than those in the control group on the seventh postoperative day (3.38 ± 2.27 vs. 6.14 ± 5.43; *P* = 0.038), and depression scores decreased but not significantly (Figure 4). To explore whether the effects of LEO aromatherapy differed between sexes, subgroup analyses of sleep and psychological outcomes were conducted. Among female participants, LEO significantly reduced anxiety scores compared with controls (3.12 ± 1.96 vs. 7.04 ± 5.21; P = 0.018), while the difference in male participants did not reach statistical significance (3.71 ± 2.71 vs. 5.00 ± 5.86; P = 0.312). Similarly, LEO appeared to improve total sleep duration and deep sleep more prominently in females than in males, although these differences did not reach statistical significance in sex-specific subgroups due to limited sample size. No significant sex-specific differences were found in depression scores, postoperative delirium, or cognitive function at follow-up (Supplementary Figure S1). The results of postoperative complications in the two groups are shown in Supplementary Table S2. No statistically significant difference was found between the two groups in the comprehensive complication index calculated by postoperative complications in the first 7 days after surgery. In addition, no significant differences in other clinical outcomes between the two groups were shown in our study (Table 3).

**TABLE 3:** Clinical outcomes between Control Group and Experimental Group.

Variables	Control group (n = 21)	Experimental group (n = 21)	χ^2^/t	P
CRP	9.55 (4.71, 29.85)	22.07 (4.13, 39.18)	−0.390	0.697
NLR	11.61 (5.31, 23.24)	11.31 (4.34, 19.76)	−0.164	0.870
Postoperative pain				
NRS-1	3.00 (2.00, 4.00)	2.00 (0.00, 4.00)	−0.835	0.404
NRS-3	1.00 (0.00, 2.00)	0.00 (0.00, 2.00)	−1.393	0.164
Postoperative nausea and vomiting				
NRS-1	0.00 (0.00, 1.00)	0.00 (0.00, 2.50)	−0.458	0.647
NRS-3	0.00 (0.00, 0.00)	0.00 (0.00, 1.00)	−0.698	0.485
Comprehensive Complication Index	21.80 ± 15.34	25.01 ± 16.60	−0.653	0.518
Postoperative length of stay	10.00 (8.00, 18.00)	11.00 (9.00, 16.50)	−0.431	0.667
ADL	80.00 (75.00, 87.50)	80.00 (70.00, 90.00)	−0.065	0.948
Cost of hospitalization	103,652.00 (92,873.00, 136,063.50)	107,589.00 (96,566.00, 124,081.00)	−0.365	0.715
PSQI-3month	8.14 ± 4.50	6.57 ± 4.00	1.197	0.238
ADL-3month	80.00 (72.50, 90.00)	90.00 (80.00, 90.00)	−0.530	0.596

Notes: Abbreviations: CRP, C-reactive protein; NLR, neutrophil-lymphocyte ratio; NRS, numerical rating scale; ADL, activities of daily living.

## Discussion

The term “lavender” originates from ancient times and is derived from the Latin word *lavare*, meaning “to wash” or “to bathe.” Lavender is a valuable aromatic plant widely cultivated for the extraction of essential oils (Vairinhos and Miguel, 2020). LEO exhibits notable biological activities, including antibacterial and antioxidant properties, and has broad applications in the medical, cosmetic, and food industries (Sattayakhom et al., 2023; Adaszy et al., 2023; Diass et al., 2023). The LEO used in the present study was characterized by high concentrations of linalool and linalyl acetate, and low levels of eucalyptol and camphor, conforming to the specifications outlined in the International Standard ISO 3515:2002. In addition, farnesene, although not monitored by ISO 3515:2002, is a very important microconstituent for the lavender essential oil flavor (Rostamkalaei et al., 2023). In the study, farnesene (1.55%) and caryophyllene (3.51%) are the main sesquiterepene hydrocarbons found in LEO.

In addition, postoperative sleep disturbance is associated with postoperative recovery, immune function and inspiratory muscle endurance, neurological outcomes, and hospitalization satisfaction (Seid Tegegne and Fenta Alemnew, 2022). Therefore, it is urgent to find a method to improve the quality of postoperative sleep, which is of great significance for improving the prognosis of patients. Postoperative sleep quality plays a crucial role in improving patient prognosis. LEO has shown in a rat model to have effects similar to diazepam, enhancing energy in the frontal and parietal lobes within 30–60 min after administration, while significantly reducing wake episodes and shortening latency to REM sleep (Manor et al., 2021). In line with previous research, the present study found that LEO improved objective postoperative sleep parameters, particularly total sleep duration, deep sleep duration, sleep latency, apnea-hypopnea index, and frequency of awakenings. Additionally, prior studies have demonstrated that inhalation of lavender aroma promotes relaxation, positively influencing physiological markers by reducing adrenal cortisol secretion, lowering sympathetic nervous system activity, and increasing parasympathetic nervous system activity (Lytle et al., 2014; Perry et al., 2012). The mechanism by which LEO improves sleep may involve the actions of its primary components, linalyl acetate and linalool, on receptors in the central nervous system, including GABA receptors and those in the limbic system (Milanos et al., 2017).

Most sleep studies have focused on subjective sleep quality, and they often assessed through tools, such as sleep diaries or the PSQI. However, only a limited number of studies have measured objective sleep parameters, such as sleep duration, sleep latency, and sleep efficiency (Wang et al., 2017; Li et al., 2021). Hospitalized patients may experience communication challenges due to the nature of their illness or the impact of various medical interventions, which can reduce the accuracy and reliability of self-reported sleep assessments. Wearable sleep monitoring devices are increasingly recognized and used in both home and clinical research settings due to their ease of use and high accuracy. Previous studies comparing the accuracy of the sleep monitor used in this study with polysomnography, the gold standard in sleep monitoring, have demonstrated good accuracy (Kurihara and Watanabe, 2012; Ding et al., 2022).

Previous studies have confirmed that sleep quality is negatively correlated with the incidence of delirium, and sleep intervention is effective in reducing the incidence and duration of delirium (Leung et al., 2015). Moreover, LEO can stimulate the olfactory bulb of the brain through the olfactory nerve, affecting the activity of the cerebral cortex, thereby improving cognitive function (Moss et al., 2003). Besides, LEO could improve the memory function through the intracranial signaling mechanisms. After the inhalation of the LEO, odorant signals travel to the secondary olfactory cortex, where the first pathway reaches and stimulates the hippocampus related to memory and the second pathway reaches the amygdala related to emotion (Ebihara et al., 2021). A study investigating the protective effects of LEO and its primary component, linalool, against cognitive deficits induced by D-galactose and aluminum trichloride in mice found that LEO, particularly linalool, could be a potential agent for improving cognitive impairment. The underlying mechanisms include LEO’s ability to protect against oxidative stress, support cholinergic function, and modulate the expression levels of proteins in the Nrf2/HO-1 pathway, as well as enhance synaptic plasticity (Xu et al., 2017). Notably, AD, a neurodegenerative disorder characterized by cognitive decline, can be mitigated by linalool, which protects against amyloid-β neurotoxicity, thereby preserving cognitive function (Caputo et al., 2021). In the current study, LEO was found to improve postoperative delirium, likely due to the neuromodulatory effects of lavender components and their interaction in regulating both sleep and cognitive function.

Surgical trauma associated with intracranial tumors is significant, leading to a prolonged recovery period and high medical costs, which undoubtedly impose a substantial psychological burden on patients during hospitalization. Patients suffering from anxiety or anxiety-related disorders often experience poor sleep quality, with sleep disturbances, particularly insomnia, being common (Johnson et al., 2006). Conversely, inadequate sleep can trigger or exacerbate anxiety (Seo et al., 2021). LEO has traditionally been used and is approved by the European Medicines Agency (EMA) as an herbal remedy for relieving stress and anxiety (Lopez et al., 2017). Numerous studies have explored the anti-anxiety effects of LEO, confirming its efficacy in alleviating anxiety (Yoo and Park, 2023). The results of exploratory subgroup analyses suggest that the anxiolytic and sleep-promoting effects of LEO may be more remarkable in female patients. This finding aligns with previous research (Ebrahimi et al., 2021) reporting greater responsiveness to aromatherapy in women, possibly due to sex-related differences in olfactory sensitivity and neuroendocrine function. However, due to the limited sample size, these results should be interpreted cautiously and warrant further investigation in larger cohorts.

One noteworthy finding of this study was that significant differences in sleep quality parameters were only observed on the fourth postoperative night. This pattern did not appear to be due to external clinical factors, as no changes in medication administration or hospital routine occurred on that day. We hypothesize that the effect of lavender essential oil may be cumulative, with several nights of continuous exposure needed to elicit measurable improvements in sleep architecture. Moreover, the fourth postoperative day often coincides with a period of relative physiological stabilization and reduced pain or surgical stress, potentially amplifying the response to the intervention. Further research with larger samples and mechanistic investigations are needed to better understand this temporal pattern of aromatherapy efficacy.

In this study, no adverse events or LEO-related complications were found during the 7-day postoperative period, indicating that short-term inhalation of 10% LEO delivered via nasal patches is likely safe for postoperative patients. This finding aligns with previous reports supporting the tolerability of lavender aromatherapy when administered in appropriate concentrations and durations. However, it is noteworthy that the safety of LEO can vary based on route of administration, dosage, and patient-specific factors. While topical and inhalational use is generally considered safe, some users may experience mild adverse reactions such as allergic dermatitis or photosensitivity (Posadzki et al., 2012; Dantas et al., 2022). In contrast, oral ingestion has been associated with potential adverse effects, including nausea, vomiting, and neurologic symptoms, particularly in children (Martin-Banderas et al., 2023; Ford et al., 2001). More notably, LEO has been implicated in hormonal disruption, especially in prepubertal boys, due to its estrogenic and antiandrogenic activity demonstrated in both *in vitro* studies and case reports (Endocrine Society, 2018; Henley et al., 2007). A 2007 report in the New England Journal of Medicine detailed cases of prepubertal gynecomastia in boys linked to repeated topical exposure to LEO, which resolved after discontinuation (Henley et al., 2007). Additional case series and laboratory data support these findings, suggesting that repeated exposure to LEO may influence endocrine function (Ramsey et al., 2019). However, some recent studies have questioned the extent of real-world hormonal impact, citing limited systemic absorption and a lack of consistent epidemiological evidence (Karpinska and Szliszka, 2023; Hawkins et al., 2022). Nevertheless, concerns about the endocrine-disrupting potential of LEO are not limited to oral ingestion. Topical application and inhalation may also pose risks, particularly in the absence of rigorous chemical characterization and dose standardization across products. Therefore, clinical use of LEO should be approached with caution across all administration routes, including oral, topical, and inhalational, especially given the lack of standardized chemical characterization. Further safety evaluation, including long-term monitoring in larger and more diverse patient populations, is warranted.

The complete chemical profile of lavender essential oil, particularly the concentrations of linalool, linalyl acetate, and minor constituents such as terpenes and sesquiterpenes, is highly relevant to its safety regardless of administration method. The variability in constituent ratios, which can result from differences in lavender species, geographic origin, harvesting conditions, and extraction methods, can significantly influence both efficacy and toxicity (Cavanagh and Wilkinson, 2002; Pavela and Benelli, 2016). For instance, studies highlighting endocrine-disrupting effects attributed these to specific constituents such as linalool and linalyl acetate, which demonstrated estrogenic and antiandrogenic activity *in vitro* (Henley et al., 2007). However, without detailed chemical characterization in those case studies, the exact exposure levels and compound interactions remain unclear, limiting the reproducibility and generalizability of the findings (Ramsey et al., 2019). Therefore, safety assessments of LEO, whether ingested, applied topically, or inhaled, must account for the compositional complexity and batch-to-batch variability of commercial products.

This study has several limitations. Firstly, the sample size was relatively small and drawn from a single center, which might limit the generalizability of the findings. Secondly, while the control group did not receive any intervention, a placebo or sham intervention was not employed, which might introduce performance or expectation bias. Thirdly, although objective sleep parameters were assessed using a dedicated sleep device, subjective sleep quality and psychological outcomes were largely based on self-reported scales, which could be susceptible to reporting bias. Fourthly, the chemical composition of the LEO used in this study was specific to *Lavandula angustifolia* ‘Jingxun 2’, and results might not be generalizable to other varieties or preparations of lavender oil. Lastly, the follow-up for cognitive outcomes was conducted via telephone, which might be less reliable than in-person assessment, particularly in detecting subtle cognitive changes.

## Conclusion

In conclusion, this is the first study to investigate the effects of LEO on postoperative sleep quality in patients with intracranial tumors. The results showed that LEO could effectively improve postoperative sleep quality, particularly on the fourth postoperative day. Additionally, it exhibited a positive impact on alleviating PNDs, as evidenced by a reduction in the duration of postoperative delirium. Furthermore, lavender aromatherapy was found to reduce anxiety scores by the seventh postoperative day. These findings hold significant implications for clinicians seeking to improve perioperative sleep, mitigate cognitive impairment, and manage stress using LEO.

## Data Availability

The original contributions presented in the study are included in the article/Supplementary Material, further inquiries can be directed to the corresponding authors.
