# Supplementary material for: Effects of lavender essential oil inhalation aromatherapy on postoperative sleep quality in patients with intracranial tumors: a randomized controlled trial
Source: Front Pharmacol. 2025 Aug 4;16:1584998. doi: 10.3389/fphar.2025.1584998 (PMC12358394; doi:10.3389/fphar.2025.1584998)
Supplement: Supplementary file 1 [file DataSheet1.docx]

Table S1 Volatile Compounds from LEO

| **Number** | **Compound** | **Empirical formula** | **RT** | **RI ^a^** | **RI ^b^** | **CAS** | **Concentration (%)** |
| --- | --- | --- | --- | --- | --- | --- | --- |
| 1 | α-Thujene | C10H16 | 6.14 | 927.84 | 929 | 2867-05-2 | 0.08 5±0.001 |
| 2 | α-Pinene | C10H16 | 6.33 | 934.64 | 937 | 80-56-8 | 0.20 ±0.0075 |
| 3 | Camphene | C10H16 | 6.74 | 949.50 | 952 | 79-92-5 | 0.23±0.0024 |
| 4 | β-Thujene | C10H16 | 7.43 | 974.24 | 966 | 28634-89-1 | 0.05±0.0023 |
| 5 | Sabinen | C10H16 | 7.52 | 977.70 | 974 | 3387-41-5 | 0.07 ±0.0044 |
| 6 | 1-Octen-3-ol | C8H16O | 7.56 | 978.99 | 980 | 3391-86-4 | 0.18 ±0.0024 |
| 7 | 3-Octanone | C8H16O | 7.78 | 986.94 | 986 | 106-68-3 | 0.17±0.004 |
| 8 | β-Myrcene | C10H16 | 7.91 | 991.65 | 991 | 123-35-3 | 0.41±0.0169 |
| 9 | α-Phellandrene | C10H16 | 8.30 | 1005.50 | 1005 | 99-83-2 | 0.06±0.0028 |
| 10 | 3-Carene | C10H16 | 8.46 | 1011.29 | 1011 | 13466-78-9 | 0.61±0.0102 |
| 11 | Hexyl acetate | C8H16O2 | 8.54 | 1014.35 | 1011 | 142-92-7 | 0.18±0.0018 |
| 12 | o-Cymene | C10H14 | 8.79 | 1023.20 | 1022 | 527-84-4 | 0.09±0.0029 |
| 13 | p-Cymene | C10H14 | 8.86 | 1025.72 | 1025 | 99-87-6 | 0.22±0.0047 |
| 14 | β-Phellandrene | C10H16 | 8.98 | 1030.14 | 1031 | 555-10-2 | 1.04±0.0268 |
| 15 | Eucalyptol | C10H18O | 9.05 | 1032.55 | 1032 | 470-82-6 | 0.84±0.0092 |
| 16 | β-Ocimene | C10H16 | 9.23 | 1039.24 | 1037 | 13877-91-3 | 5.42±0.0957 |
| 17 | trans-β-Ocimene | C10H16 | 9.52 | 1049.50 | 1049 | 3779-61-1 | 2.45±0.0207 |
| 18 | γ-Terpinene | C10H16 | 9.81 | 1060.11 | 1060 | 99-85-4 | 0.05±0.0036 |
| 19 | cis-Sabinene hydrate | C10H18O | 10.06 | 1068.96 | 1070 | 15537-55-0 | 0.04±0.0002 |
| 20 | Linalool oxide | C10H18O2 | 10.21 | 1074.50 | 1074 | 5989-33-3 | 0.07±0.0007 |
| 21 | Isoterpinolene | C10H16 | 10.58 | 1087.55 | 1086 | 586-63-0 | 0.04±0.0008 |
| 22 | Terpinolene | C10H16 | 10.64 | 1089.75 | 1088 | 586-62-9 | 0.12±0.0052 |
| 23 | Linalool | C10H18O | 10.98 | 1102.19 | 1099 | 78-70-6 | 27.85±0.4082 |
| 24 | 1-Octen-3-yl-acetate | C10H18O2 | 11.27 | 1113.15 | 1111 | 2442-10-6 | 1.19±0.0097 |
| 25 | E-p-Mentha-2,8-dienol | C10H18O | 11.53 | 1123.30 | 1123 | 7212-40-0 | 0.04±0.0030 |
| 26 | Neo-allo-ocimene | C10H16 | 11.72 | 1130.53 | 1131 | 7216-56-0 | 0.17±0.0052 |
| 27 | (+)-2-Bornanone | C10H16O | 12.15 | 1147.14 | 1143 | 464-49-3 | 0.16±0.0020 |
| 28 | Hexyl isobutyrate | C10H20O2 | 12.23 | 1150.13 | 1150 | 2349-07-7 | 0.03±0.0056 |
| 29 | endo-Borneol | C10H18O | 12.70 | 1168.20 | 1167 | 507-70-0 | 2.42±0.0502 |
| 30 | (3E,5Z)-1,3,5-Undecatriene | C11H18 | 12.89 | 1175.47 | 1174 | 51447-08-6 | 0.14±0.0064 |
| 31 | L-terpinen-4-ol | C10H18O | 12.99 | 1179.58 | 1182 | 20126-76-5 | 0.91±0 .0182 |
| 32 | m-Cymen-8-ol | C10H14O | 13.09 | 1183.43 | 1180 | 1197-01-9 | 0.07±0.0044 |
| 33 | p-Cymen-8-ol | C10H15O | 13.18 | 1186.74 | 1183 | 1197-01-9 | 0.04±0.0060 |
| 34 | Crypton | C9H14O | 13.23 | 1188.50 | 1184 | 500-02-7 | 0.26±0.0053 |
| 35 | α-Terpineol | C10H18O | 13.33 | 1192.46 | 1189 | 98-55-5 | 0.85±0.0177 |
| 36 | cis-Geraniol | C10H18O | 14.24 | 1229.72 | 1228 | 106-25-2 | 0.06±0.0236 |
| 37 | p-Cumic aldehyde | C10H12O | 14.56 | 1243.05 | 1239 | 122-03-2 | 0.09±0.0482 |
| 38 | D-Carvone | C10H14O | 14.66 | 1247.03 | 1246 | 2244-16-8 | 0.03±0.0010 |
| 39 | Linalyl acetate | C12H20O2 | 14.95 | 1259.19 | 1257 | 115-95-7 | 34.50 ±0.3183 |
| 40 | Isopulegol acetate | C12H20O2 | 15.47 | 1280.78 | 1285 | 57576-09-7 | 0.07±0.0092 |
| 41 | Bornyl acetate | C12H20O2 | 15.67 | 1289.21 | 1285 | 76-49-3 | 0.15±0.0018 |
| 42 | Lavandulol acetate | C12H20O2 | 15.75 | 1292.49 | 1287 | 25905-14-0 | 10.74 ±0.1494 |
| 43 | Car-3-en-5-one | C10H14O | 16.28 | 1315.56 | 1314 | 81800-50-2 | 0.03±0.0029 |
| 44 | Nerol acetate | C12H20O2 | 17.42 | 1365.94 | 1364 | 141-12-8 | 0.18±0.0027 |
| 45 | Geranyl acetate | C12H20O2 | 17.84 | 1384.79 | 1382 | 105-87-3 | 0.31±0.0584 |
| 46 | 7-epi-Sesquithujene | C15H24 | 18.04 | 1393.51 | 1391 | 159407-35-9 | 0.03 ±0.0036 |
| 47 | cis-α-Bergamotene | C15H24 | 18.60 | 1419.84 | 1415 | 18252-46-5 | 0.05±0.0032 |
| 48 | Caryophyllene | C15H24 | 18.74 | 1426.59 | 1419 | 87-44-5 | 3.51±0.0886 |
| 49 | trans-α-Bergamotene | C15H24 | 19.03 | 1440.53 | 1435 | 13474-59-4 | 0.16±0.0040 |
| 50 | α-Himachalene | C15H24 | 19.24 | 1450.33 | 1449 | 3853-83-6 | 0.04±0.0010 |
| 51 | Humulene | C15H24 | 19.30 | 1453.24 | 1454 | 6753-98-6 | 0.03±0.0011 |
| 52 | (E)-β-Famesene | C15H24 | 19.43 | 1459.32 | 1457 | 18794-84-8 | 1.55±0.0450 |
| 53 | epi-β-Caryophyllene | C15H24 | 19.52 | 1463.42 | 1466 | 68832-35-9 | 0.04±0.0028 |
| 54 | Germacrene D | C15H24 | 20.04 | 1488.34 | 1481 | 23986-74-5 | 0.65 ±0.0248 |
| 55 | β-Bisabolene | C15H24 | 20.53 | 1512.62 | 1509 | 495-61-4 | 0.03 ±0.0033 |
| 56 | γ-Cadinene | C15H24 | 20.70 | 1521.05 | 1513 | 39029-41-9 | 0.21±0.0012 |
| 57 | δ-Cadinene | C15H24 | 20.78 | 1524.84 | 1524 | 483-76-1 | 0.02 ±0.0012 |
| 58 | cis-Calamenene | C15H22 | 20.88 | 1529.88 | 1531 | 72937-55-4 | 0.03±0.0021 |
| 59 | Caryophyllene oxide | C15H24O | 22.12 | 1592.68 | 1581 | 1139-30-6 | 0.30 ±0.0051 |
| 60 | tau.-Cadinol | C15H26O | 23.19 | 1648.88 | 1640 | 5937-11-1 | 0.14 ±0.0032 |
|  | Monoterpenes |  |  |  |  |  | 92.11±0.1996 |
|  | Sesquiterpenes |  |  |  |  |  | 6.78±0.1764 |
|  | Others |  |  |  |  |  | 0.79±0.0097 |
|  | Total |  |  |  |  |  | 99.68 ±0.0443 |

The numbers are arranged according to retention time, and values (relative peak area percent) represent averages of three determinations; RI^a^: calculated retention index of standard mixture of n-alkanes on HP-5MS capillary column; RI^b^: retention index of the component on semi-standard non-polar capillary column from NIST and reported literature.

Table S2 Classification of postoperative complications based on the Clavien-Dindo classification

| Postoperative Complications | Control Group | Experimental Group |
| --- | --- | --- |
| Grade Ⅰ |  |  |
| Fever (T > 38.5 C) | 3 (6.8%) | 3 (6.7%) |
| Electrolyte disorders | 5 (11.4%) | 11 (24.4%) |
| Postoperative nausea and vomiting | 9 (20.5%) | 5 (11.1%) |
| Grade Ⅱ |  |  |
| Coagulation disorders | 2 (4.5%) | 6 (13.3%) |
| Hypoproteinemia | 1 (2.3%) | 2 (4.4%) |
| Intracranial infections | 1 (2.3%) | 0 (0%) |
| Pneumonia | 6 (13.6%) | 5 (11.1%) |
| Venous thrombosis | 7 (15.9%) | 5 (11.1%) |
| Seizure | 2 (4.5%) | 0 (0%) |
| Delirium | 5 (11.4%) | 4 (8.9%) |
| Cerebral infarction | 2 (4.5%) | 0 (0%) |
| Grade Ⅲ b |  |  |
| Intracranial infections | 0 (0%) | 1 (2.2%) |
| Grade Ⅳ a |  |  |
| Single organ dysfunction | 1 (2.3%) | 3 (6.7%) |


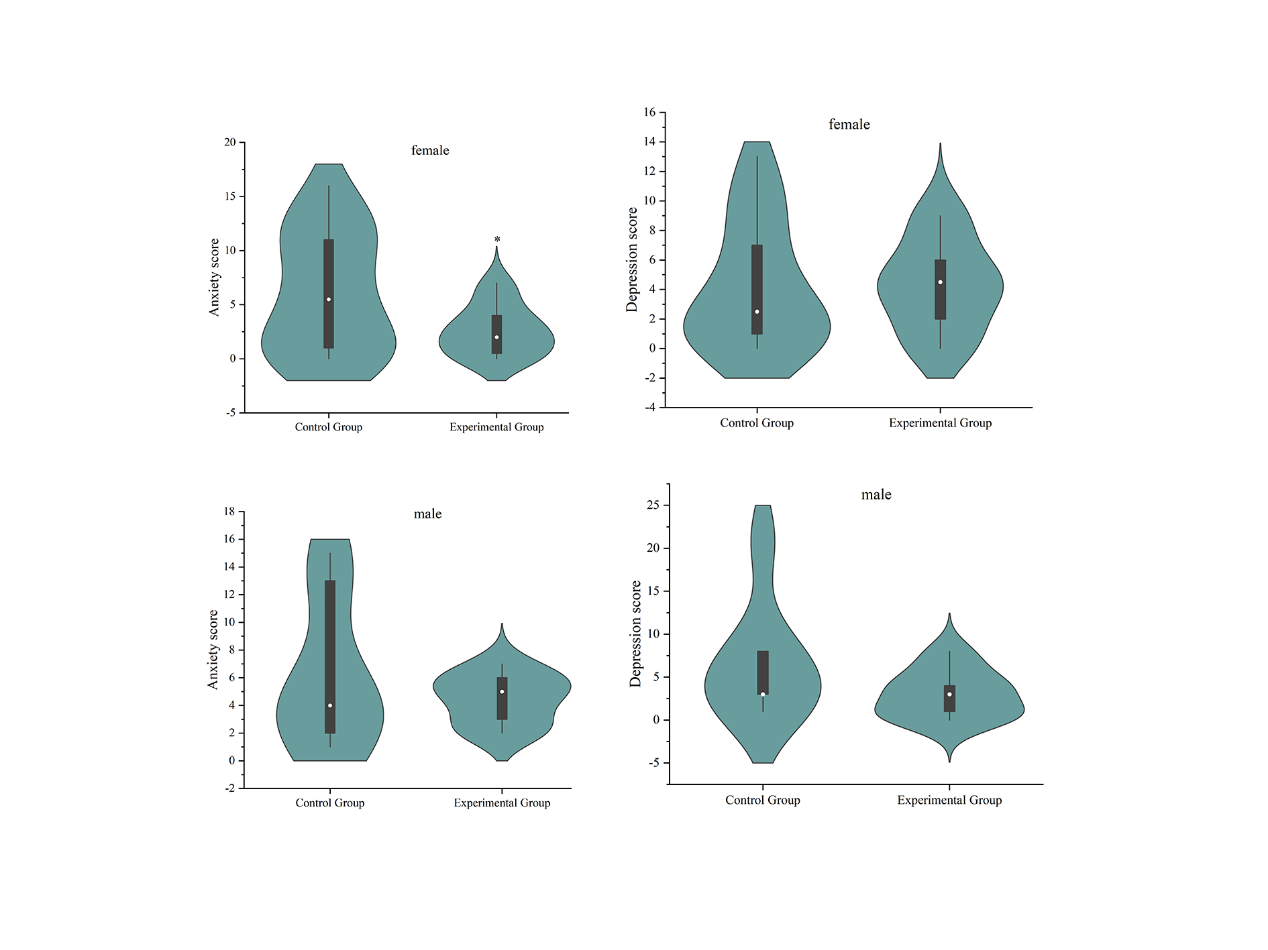


Figure S1 Analysis of anxiety score and depression score in two groups based on gender. Values shown are mean ± SE of twenty-one replicates. Asterisk (*) indicates significant differences (**P* < 0.05).
